# Testosterone-receptor positive hepatocellular carcinoma in a 29-year old bodybuilder with a history of anabolic androgenic steroid abuse: a case report

**DOI:** 10.1186/s12876-015-0288-0

**Published:** 2015-05-20

**Authors:** Philipp Solbach, Andrej Potthoff, Hans-Jürgen Raatschen, Bisharah Soudah, Ulrich Lehmann, Andrea Schneider, Michael J. Gebel, Michael P. Manns, Arndt Vogel

**Affiliations:** 1 Department of Gastroenterology, Hepatology and Endocrinology, Medizinische Hochschule, OE 6810 Carl-Neuberg-Str. 1, 30625 Hannover, Germany; 2 Department of Diagnostic and Interventional Radiology, Medizinische Hochschule Hannover, Hannover, Germany; 3 Department of Pathology, Medizinische Hochschule Hannover, Hannover, Germany

**Keywords:** Anabolic androgenic steroids, Hormonal treatment, Hepatocellular carcinoma, Hepatic adenoma

## Abstract

**Background:**

Continuous use of anabolic androgenic steroid in high-doses is associated with substantial health risks, including hepatocellular adenoma. Malignant transformation from hepatocellular adenoma to hepatocellular carcinoma after anabolic androgenic steroid abuse has been rarely reported. The morphological distinction of adenoma from well-differentiated hepatocellular carcinoma is challenging and requires elaborated imaging techniques and histology.

**Case presentation:**

We report about a 29-year old male professional bodybuilder who presented with mid-epigastric pain at the emergency unit. Ultrasound showed a severe hepatomegaly with multiple lesions. Contrast-enhanced ultrasound revealed a heterogeneous pattern with signs of hepatocellular carcinoma. CT scan of the abdomen confirmed multiple hypervascular lesions and central areas of necrosis without contrast enhancement. Subsequent diagnostics included fine needle aspiration (FNA) of suspicious lesions and mini-laparoscopy to establish the diagnosis of a β-catenin and testosterone-receptor positive hepatocellular carcinoma embedded in multiple adenomas. The patient was subsequently treated by liver transplantation and remains tumor-free 27 month after surgery.

**Conclusion:**

Hepatocellular carcinoma occurring in association with anabolic androgenic steroid abuse should sensitize physicians and especially professional bodybuilders for the harmful use of high doses of steroids.

## Background

One of the major risk factors of developing hepatocellular adenoma (HCA) is the use of oral contraceptives, which stimulate liver expressed estrogen and androgen receptors, predominantly in women between 15 and 45 years of age [1, 2]. Accordingly, 90 % of HCA are diagnosed in women [3]. Other risk factors include glycogen storage disease I and III and treatment with anabolic steroids in patients with Fanconi’s anemia [4–6]. A few reports indicate that anabolic androgenic steroids (AAS) may also lead to the formation of HCA [7–9]. Relevant complications of HCA include hemorrhage and malignant transformation into hepatocellular carcinoma (HCC) depending on size and β-catenin activation.

In the literature are more reports of patients with Fanconi’s anemia, which received medical treatment with AAS and subsequently developed HCCs [4, 6]. Furthermore a few cases are described from bodybuilders with AAS abuse that subsequently developed HCC [7, 9, 10]. These patients require close surveillance to detect possible malignant transformation from HCA into HCC or lesions that are at risk of bleeding or rupture [8].

Here, we report a case of a testosterone-receptor positive HCC arising from multiple HCAs in a professional bodybuilder after 6 years of AAS abuse and the difficulties of diagnostics and therapeutical options.

## Case presentation

### Clinical history

We describe a case of a 29-year old male professional bodybuilder who presented at the emergency unit with midepigastric-pain. He had been taking anabolic androgenic steroids (AAS; see below) and underwent strict nutritional diets to increase muscle mass prior to competitions over the last 6 years. He self-administered the following AAS from 2011 to 2012 in cycles of 4 weeks with rest periods of several weeks between the cycles: nandrolone decanoate (400 mg/week), sustanon (750 mg/week), methandienone (280–350 mg/week), stanozolol (50 mg/day for 1 month) and human growth hormone (4 IE/day for 3 month). Additionally 3 days before competitions he self-administered the diuretics aldosterone (50 mg/day) and thiazide (25 mg/day) to reduce extracellular and subcutaneous tissue volume and to achieve a better muscle shaping. Furthermore insulin injections and tamoxifen were administered. The frequency of self-administration was varying in each cycle.

**Fig. 1:**
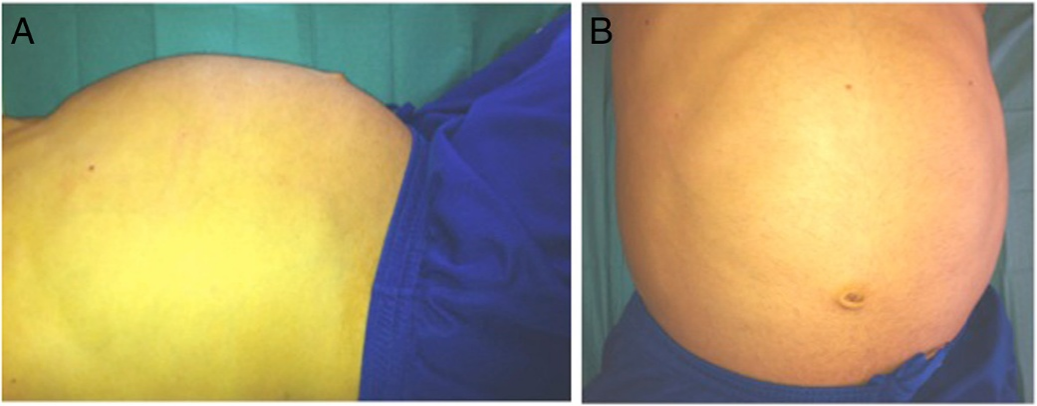
Clinical examination showed a muscular body, height 186 cm, weight 120 kg. The lung showed a sonorous percussion, vesicular breath sounds, no noise. Heart: regular rhythm, 55 bpm, RR 134/67 mmHg, first heart sound and second heart sound not flashy, no heart murmurs. No pathological examination findings for head, neck, lymph node status, pulse status. No scleral icterus. Abdominal examination showed a large abdomen, vascular abdominal markings, yellow colouring, liver palpable to lesser pelvis right, superficial palpation was painful, spleen not palpable, no kidney pain or back pain (**a** lateral view; **b** ventral view)

At presentation, the patient had not taken any AAS for 5 weeks. Previous history was a childhood near a nuclear power plant until the age of fifteen. Allergic coryza and nasal spray use since the age of seven were reported. Moreover no abuse of ethanol or smoking was given. The patient’s father died supposedly as the result of kidney cancer, his grandfather died from bronchial cancer. His mother and younger siblings are healthy. On examination, the patient showed a three-fold amplified liver extending into lesser pelvis with painful palpation. No other clinical abnormalities were detectable (Fig. 1).

### Laboratory findings

Laboratory evaluation revealed severe hepatic inflammation and an impaired liver function (ALT 1653 IU/l; AST 1437 IU/l; alkaline phosphatase 372 IU/l; GGT 463 IU/l; CHE 1.29 kU/I; LDH 695 U/I; total bilirubin 41 μmol/l; direct bilirubin 38 μmol/l). Hematology showed an anemia and slightly increased white blood cell count (leucocytes 12.500/ μl; hemoglobin 8.5 g/dl; INR 1.04; reticulocytes 104/nl). Inflammation parameters were increased (CRP 81 mg/l, PCT 4.8 μg/l, Ferritin 1672 μg/l). The renal function was almost normal (creatinine 123 μmol/l, but cystatine c 0.76 mg/l and MDRD > 60 ml/min).

Coagulation tests were normal, as well as hepatitis virus markers, including hepatitis A, B and C and also human deficiency virus markers were negative. Serum levels of sex hypophyseal hormones (LH <0.07 U/I, FSH <0.3 U/I, androgens (T + DHT) 0.037 ng/ml, free-testosterone 4.94 pg/ml) and thyroid gland markers (fT3, fT4) were below the lower limit of normal. This constellation of sex hormones reflects anabolic steroids induced hypogonadism (ASIH).

### Imaging

**Fig. 2:**
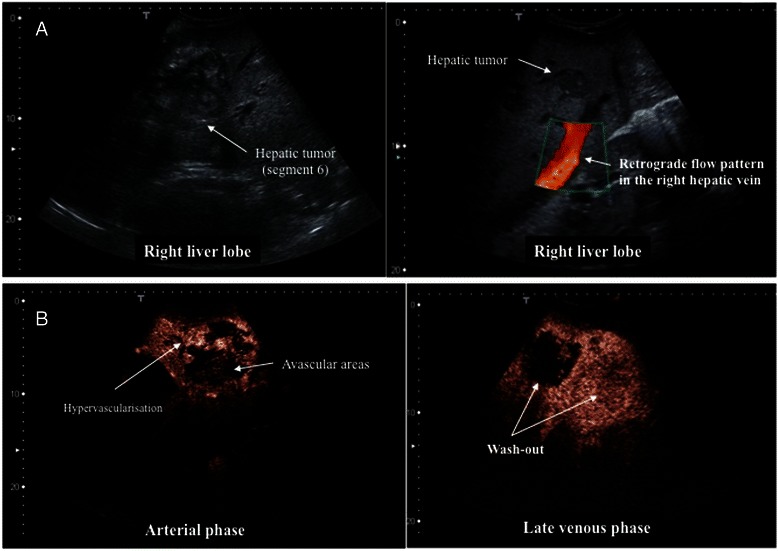
**a** B-mode ultrasonography: The hepatic tumors were polycyclic, isoechoic and inhomogeneous. Doppler sonography showed a compression in the IVC hepatic segment by the liver tumors with consecutive development of partial Budd Chiari syndrome diagnosed by the retrograde flow pattern in the right hepatic vein and the presence of subcapsular venous collaterals. **b** Contrast enhanced ultrasonography (CEUS): CEUS was performed using a bolus injection of 1.5 mL SonoVue® (Bracco SpA, Milan, Italy). The largest lesion showed a large, avascular center with hypervascular margin in the arterial phase. In the late venous phase parts of the center and the margin showed wash-out

Abdominal ultrasound (US) showed a marked hepatomegaly with 25.6 cm in the midclavicular line and 30.1 cm in the median with evidence of fatty liver. In B-mode sonography multiple lesions were detected in both liver lobes (left liver lobe: various isoecogenic, inhomogeneous lesions with a maximum size of 92 mm × 92 mm; right liver lobe: various isoecogenic, polycyclic inhomogeneous lesions with a maximum size of 222 mm × 162 mm) (Fig. 2a). Moreover, the hepatic segment of the inferior vena cava (IVC) was compressed by the hepatic tumors, which consecutively led to a partial Budd Chiari syndrome. This was diagnosed in the doppler sonography through the retrograde flow pattern in the right hepatic vein with an evidence of subcapsular venous collaterals.

**Fig. 3:**
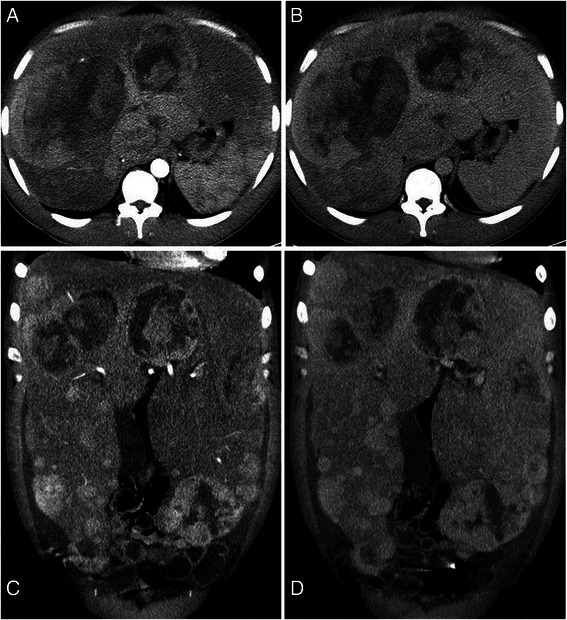
Contrast enhanced computed tomography in arterial (**a**,**c**) and portalvenous phase (**b**,**d**): Highly enlarged liver with curved borders secondary to multiple parenchymatous lesions throughout all liver segments, with arterial enhancement and no substantial wash-out during portalvenous phase. The large lesion in right lobe up to 16 cm appeared inhomogeneous with peripheral enhancement and non-enhancing, necrotic areas in the center. Intrahepatic vena cava and right liver vein were completely compressed by nodular tumors in the caudate lobe and the large lesion in the right lobe

In contrast-enhanced ultrasound (CEUS) the hepatic lesions showed a heterogeneous pattern (Fig. 2b). Some lesions exhibited arterial enhancement, initially at the periphery with subsequent very rapid centripetal filling and without wash-out in the portal venous or late phase. Other lesions showed arterial enhancement, with a chaotic vascular pattern and avascular areas in different parts of each tumor. In the portal venous phase, these tumors showed wash-out, especially in liver segment 6, in which a tumor biopsy was performed. The small lesions (diameter <12 mm) seen in B-mode sonography showed neither arterial hypervascularisation nor wash-out in the portal venous or late phase. The contrast-enhanced CT scan of the abdomen revealed a distinctive hepatomegaly with multiple hypervascular lesions without substantial wash-out in portal venous phase. In the largest lesions, central areas of necrosis without contrast enhancement were present (16 × 13 cm) (Fig. 3). There was no evidence of metastasis in the abdomen and chest.

### Pathology

Cytology of liver-segment six was performed by fine needle aspiration (FNA) of one lesion with signs of hepatocellular carcinoma in contrast-enhanced ultrasound. The cytological samples did not reveal malignancy and showed no clearly signs for hepatocellular carcinoma or hepatoblastoma. Further molecular cytogenetic and pathological-anatomical diagnostics showed atypical HCA.

**Fig. 4:**
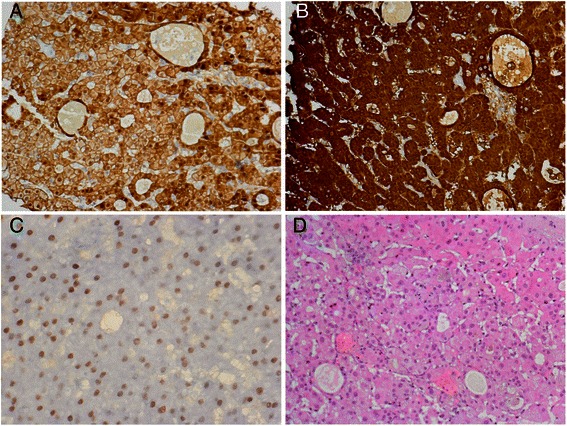
Liver biopsy: Small solid and glandular associations of hepatocytes without bile duct epithelia. Reticulin fiber network focally obtained. The hepatocytes had markedly enlarged nuclei with large nucleoli with coarse structure of chromatin. Iron and rhodanine negative. Immunohistochemistry: Beta-catenin 20 % nuclear positive (**a**; 80:1) and glutamine synthetase cytoplasmic positive (**b**; 160:1), hepatocytes with nuclear positivity for androgen-receptors (**c**; 140:1). Molecular Cytogenetics: Aneuploidy of chromosome 1 (CEP1) and 8 (CEP). Diagnosis: The results of conventional histology, molecular biology and immunohistochemistry recommended HCC G1 (**d**; HE stain 140:1). Comment: Simply by Cytology (FNP) a detection of HCC G1 is quite difficult. There were no bridges or cell atypia, on the other hand, histology showed more criteria for HCC G1

To confirm the diagnosis of hepatocellular carcinoma in suspicious lesions, a diagnostic laparoscopy was performed. The liver was enormously enlarged and there was almost no veritable parenchyma visible between the tumor nodules. The largest tumors bulged out of the liver’s surface with yellow to bluish discoloration and strong tumor vascular markings. Biopsy of the liver in the right lobe from three tumors and one tumor of the left lobe was performed. The synopsis of morphology and immunohistochemistry now clearly confirmed the diagnosis of a hepatocellular carcinoma (Fig. 4d; β-catenin 20% nuclear positive (Fig. 4a), glutamine synthetase cytoplasmic positive (Fig. 4b), androgen-receptor nuclear positive (Fig. 4c) and focal CD34 positive). Sequence analysis of exon 3 of the β-catenin gene was performed by PCR and subsequent Sanger sequencing as described in reference eleven and showed a hotspot mutation in codon 32 (p.D32G) [11].

### Treatment

Due to the size and number of the lesions, there was no option for surgical resection. The distinction of all tumor nodules into HCAs and hepatocellular carcinomas was impossible, but overall HCC burden was considered to be out of the Milan criteria [12]. The multidisciplinary tumor board at Medical School Hannover initially recommended chemoembolization, which was however declined by the patient. He therefore returned home, where subsequently a liver transplantation was performed without any complications. The explanted liver showed a mass of 8 kg. 27 months after transplantation he is in excellent condition without any signs of metastasis or local recurrence. He is not taking AAS anymore.

### Discussion

We report a case of a young professional bodybuilder with self-administration of high-doses of anabolic androgenic steroids (AAS) for at least 6 years who developed a hepatocellular carcinoma (HCC) without metastasis or alpha-fetoprotein elevation. In this case, the hepatocellular carcinoma was embedded in multiple hepatocellular adenomas (HCA).

AAS such as testosterone and its derivates are favored in endurance and strength sports as well as in bodybuilding, often in combination with other medication like diuretics and insulin. The most popular oral products especially stanozolol, methandrostenolone and nandrolone have a high first-pass in the liver and have been reported to induce significant toxicity including intrahepatic structural changes with cholestasis and benign and malignant tumors.

It has been reported, that steroids induce HCA with potential malignant transformation into carcinoma. As described for colorectal-carcinoma, there could be an adenoma-carcinoma sequence [13]. In our case, it is difficult to distinguish whether the HCC developed de novo or within the multiple HCAs. Several biopsies from multiple tumor nodules were required to ascertain the diagnosis of HCC. The morphological distinction of HCA, focal nodular hyperplasia (FNH), macroregenerative nodules from well-differentiated HCC can be challenging [14].

Malignant transformation of HCA may occur in about 4.5–9 % of cases [14–17]. Exome sequencing has identified recurrent somatic activating mutations in several genes including FRK, JAK1, gp130, and β-catenin. Moreover, integrative analysis of HCAs transformed to hepatocellular carcinoma revealed that β-catenin mutation occurs as an early alteration, whereas TERT promoter mutations are associated with the last step of the adenoma-carcinoma transition [18]. HCAs with ß-catenin mutations frequently show an overexpression of β-catenin (nuclear and cytoplasmic) and glutamine synthetase as in our patient. Although HCA shows an increased prevalence in women, beta-catenin activation is more prevalent in men with consequently an increased prevalence of HCC [19, 20]. In our case genetic analysis for β-catenin mutations showed a hotspot mutation in codon 32 of exon 3 (p.D32G).

As has been described previously, HCA showed highly variable appearance on computed tomography (CT), magnetic resonance imaging (MRI), and contrast-enhanced ultrasound (CEUS) scans. This is reflected by the differences in the histological features [21–25]. In the CEUS, typical HCA shows an arterial hypervascularisation in the early arterial phase with a centripetal filling pattern. However this arterial enhancement pattern can also be encountered in HCC and is not pathognomonic of HCA [26]. MRI seems to be superior to other imaging modalities for the diagnosis of HCA. One study performed by *Laumonier* and coworkers revealed that in hepatocyte nuclear factor 1 alpha (HNF1A) -mutated and inflammatory HCAs characteristic MRI patterns exist, which reach a specificity of 100 % and a sensitivity of 86.7 % [27]. Histology is the ultimate gold standard for the diagnosis and risk stratification of HCAs [28].

Immunochemistry analysis revealed the presence of testosterone-receptors on the hepatocellular carcinoma, which might be of prognostic significance. In one study, none of the patients with androgen receptor (AR) positive HCC survived 5 years [29]. Other studies supported these findings by suggesting a negative impact of AR positivity on tumor recurrence [30]. Various clinical trials have evaluated the role of anti-androgens for the treatment of liver cancer with controversial outcome. In a systematic review, *Di Maio* et al. concluded that hormonal treatment should not be a part of the current management of HCC patients [31, 32].

There are different therapeutic strategies for HCC without metastasis. First of all local ablative therapies like percutaneous ethanol injection (PEI), radiofrequency ablation (RFA), transarterial chemoembolization (TACE) and radiation therapy (RT) but effectiveness depends on the number and size of the tumors. Due to the tumor burden of our patient local ablative therapies such as RFA or surgery were not possible. TACE for bridging was recommended by the tumorboard, although a clear differentiation between HCA and carcinoma was not given. This minimally- invasive method can treat multiple tumors, can be easily repeated and is well established in the treatment algorithm for patients with advanced HCC [33]. Embolization of HCA has been rarely reported, but appears to be a feasible therapeutic option [34]. Our patient refused to take this option.

In general liver transplantation is the therapy of choice for selected patients with HCC without the possibility of resection and extrahepatic metastasis [35]. It is known, that patients exceeding Milan criteria for liver transplantation had a higher recurrence rate and lower survival rate than complying them. But also beyond the Milan criteria but within extended University of California, San Francisco (UCSF) criteria (single tumor <6.5 cm, maximum of three total tumors with none >4.5 cm, and cumulative tumor size <8 cm) a prolonged survival can be achieved. Survival rates beyond UCSF criteria were less than 50 % at 5 years [36]. Following liver transplantation, our patient is still without any signs of tumor recurrence or metastasis.

## Conclusion

In this case, 6 years of chronic anabolic androgenic steroid abuse lead to HCA and HCC development with almost no normal liver tissue left. Not only bodybuilders but also trainers and physicians should be aware of the HCC risk when using or prescribing AAS.

### Consent

Written informed consent was obtained from the patient for publication of this Case report and any accompanying images. A copy of the written consent is available for review by the Editor of this journal.

## Abbreviations

AAS: Anabolic androgenic steroids
ALT: Alanine transaminase
AR: Androgen receptor
AST: Aspartate transaminase
AFP: Alfa-fetoprotein
CEUS: Contrast-enhanced ultrasonography
CHE: Cholinesterase
CRP: C-reactive protein
CT: Computer tomography
FNA: Fine needle aspiration
FSH: Follicle stimulating hormone
GGT: Gamma-glutamyl transferase
HCA: Hepatocellular adenoma
HCC: Hepatocellular carcinoma
INR: International normalized ratio
IVC: Inferior vena cava
LDH: Lactate dehydrogenase
LH: Luteinising hormone
MDRD: Modification of diet in renal disease
MRI: Magnet resonance imaging
PCT: Procalcitonin
PEI: Percutaneous ethanol injection
RFA: Radiofrequency ablation
RT: Radiation therapy
TACE: Transarterial chemoembolization
